# Yield and Metabolite Production of *Pelargonium sidoides* DC. in Response to Irrigation and Nitrogen Management

**DOI:** 10.3390/metabo10060219

**Published:** 2020-05-27

**Authors:** Motiki M. Mofokeng, Gerhard Prinsloo, Hintsa T. Araya, Christian P. du Plooy, Ntshakga R. Sathekge, Stephen O. Amoo, J. Martin Steyn

**Affiliations:** 1 Agricultural Research Council, Roodeplaat-Vegetable and Ornamental Plant (ARC-VOP), Pretoria 0001, South Africa; arayah@arc.agric.za (H.T.A.); iduplooy@arc.agric.za (C.P.d.P.); amoos@arc.agric.za (S.O.A.); 2 Department of Plant and Soil Sciences, University of Pretoria, Hatfield, Pretoria 0002, South Africa; martin.steyn@up.ac.za; 3 Department of Agriculture and Animal Health, University of South Africa, Science Campus, Johannesburg 1710, South Africa; prinsg@unisa.ac.za

**Keywords:** alanine, aspartate, citrate cycle, esculin, gluconeogenesis, glutamate metabolism, medicinal plant, scopoletin, umckalin, fertilizer

## Abstract

Competition for water between agricultural and non-agricultural economic sectors hampers agricultural production, especially in water-scarce regions. Understanding crop responses in terms of yield and quality to irrigation is an important factor in designing appropriate irrigation management for optimal crop production and quality. *Pelargonium sidoides* DC., often harvested from the wild, is in high demand in the informal market and for commercial formulations. Agricultural production of high-quality materials through cultivation can help reduce pressure on its wild populations. This study aimed at determining the effects of water and nitrogen on *P. sidoides* yield and metabolite production. The irrigation treatments applied were 30%, 50%, and 70% of an allowable depletion level (ADL), while the nitrogen (N) levels were 0 (control), 50, 100, and 150 kg ha^−1^. The 30% ADL resulted in a significantly higher biomass and root yield. Nitrogen at 50 and 100 kg ha^−1^ resulted in a significantly higher biomass yield, compared to the N control. An increase in sugars and citrate cycle components was observed for the well-watered 30% ADL treatment, whereas water-stressed (50% and 70% ADL) treatments increased alanine, aspartate, and glutamate metabolism, increasing levels of asparagine, 4-aminobutyrate, and arginine. The treatments had no significant effect on the root content of esculin, scopoletin, and umckalin. Water stress induced metabolite synthesis to mitigate the stress condition, whereas under no water stress primary metabolites were synthesized. Moreover, cultivation of *P. sidoides* as a conservation strategy can increase yield without affecting its bioactivity, while providing sustenance for the rural communities.

## 1. Introduction

Agriculture is the major user of water resources in many regions of the world. With increasing aridity and a growing world population, water will become a scarcer commodity [1]. In a drought-prone country such as South Africa, allocation of water resources to other economic sectors due to competition for use may impose restrictions on agriculture, unless innovative irrigation management aimed at saving water while maximizing productivity, is introduced [2]. Understanding crop responses to lack or excess of water is a prerequisite for the development of appropriate irrigation management guidelines for optimal production [3].

Since nutrient input is directly linked to irrigation [4], the best management strategy is to integrate the two production factors. The yield and composition of secondary metabolites in medicinal plants are influenced by geographical and environmental factors [5]. Secondary metabolites are associated with increased survival, either by coping with unfavourable environmental conditions or by regulating some metabolic processes [6]. Metabolomics comprises the detection of metabolites present within a biological system under specific conditions, thus providing a phenotypic assessment of the system [7].

*Pelargonium sidoides* DC. is one of several geophytic species that are of economic importance in traditional/complementary medicines in South Africa [8]. The tuberous roots of *P. sidoides*, which the plant uses to perennate through several years, are used as a raw material for important phytomedicines and other herbal medicinal products used for the treatment of upper respiratory tract infections [9,10]. Over-exploitation of *P. sidoides* arising from the high demand for its local use and international pharmaceuticals coupled with its unsustainable harvesting by uprooting the whole plants has led to declines in its wild populations. It has been reported that in the Grahamstown area (Eastern Cape Province, South Africa), approximately 14,000 hectares of farm, municipal, and government land had been repeatedly stripped of *P. sidoides* species, beyond possibilities of re-growth and regeneration [11]. Most of the gatherers in the Eastern Cape Province are from rural communities, with no other source of income and thus they rely heavily on natural resources for sustenance [9,12]. Thus, cultivation will not only ensure conservation and sustainable use of the species, but it will also improve the livelihood of the rural harvester communities. However, these communities have limited access to other resources such as water for irrigation and fertilizers.

The medicinal properties of *P. sidoides* root extracts are ascribed to eight different coumarins, of which umckalin and 5,6,7-methoxycoumarin are useful marker compounds [13,14,15,16]. The presence of esculin and scopoletin in the root extracts of *P. sidoides* has also been reported [17,18,19,20]. *Pelargonium sidoides* has a significantly high yield of coumarins, with umckalin amounting for about 40% of the total coumarin content [16] and this could be a possible reason for preference of *P. sidoides* roots as compared to *P. reniforme*. The aim of the present study was to investigate the metabolite production and yield responses of *P. sidoides* to different levels of water and nitrogen, to provide guidelines for sustainable production of this plant.

## 2. Results and Discussion

### 2.1. Soil Water Deficits and Water Use

The water treatments and the amounts of water applied for each treatment are provided in Table 1. The total reference atmospheric evaporative demand (ETo) for the treatment period in our study was 663 mm. Similarly, it was reported that rose-scented geranium at 20% of maximum allowable depletion (MAD) recorded 476 mm ET and the 80% MAD treatment recorded 253 mm ET [2]. *Pelargonium sidoides* showed increased stomatal conductance under well-watered conditions, which explains the increased water usage observed, apart from its larger canopy, whereas under water stress the stomata were partially to fully closed, resulting in lower stomatal conductance [4].

### 2.2. Total Fresh and Dry Biomass Yield

**Figure 1:**
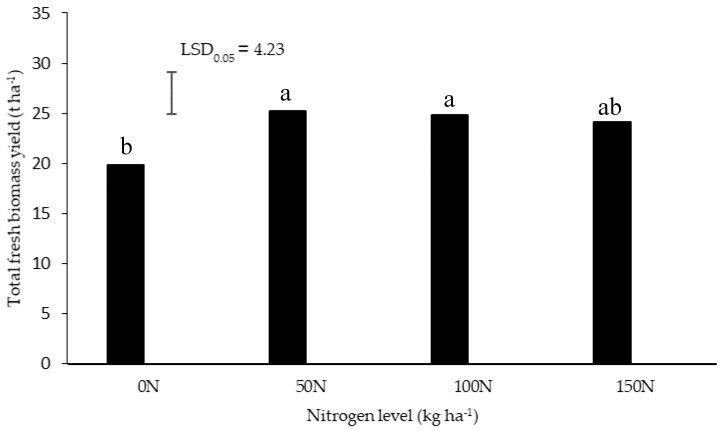
Total fresh biomass yield of *Pelargonium sidoides* in response to nitrogen level. The error bar on the figure represents Least Significant Difference (LSD), at 95% level of statistical probability. Bars with the same letter were not significantly different (*P* > 0.05).

There was no significant interaction between the N and water depletion levels on total fresh biomass yield, fresh root yield, dry root yield, and average dry matter content. The N application at 50 and 100 kg ha^−1^ significantly increased total biomass (Figure 1). No significant differences were observed between the 50, 100, and 150 kg ha^−1^ N treatments; and between the control and highest level of 150 kg ha^−1^ N. This compares well to the N requirements of other medicinal and essential oil crops. The highest total biomass for palmarosa (*Cymbopogon martinii* (Roxb.) Wats. var. motia Burk.), an essential oil crop, was produced by applying 80 kg ha^−1^ N under rainfed conditions [21]. Fertilization with 160 kg ha^−1^ N significantly increased the herbage yield of *Pelargonium graveolens*, beyond which there were no further significant yield improvements [22]. An increase in fresh herbage yield of rose-scented geranium (*Pelargonium capitatum* (L.) *L′Hér.* x *P.radens H.E. Moore*) at 100 kg ha^−1^ N compared to the control has been reported, beyond which there were no significant yield increases [23].

The well-watered treatment (30% ADL) had a significantly higher fresh biomass yield than the two water-stressed treatments (Table 1). There were no significant differences between the moderately (50% ADL) and severely stressed (70% ADL) treatments. The dry biomass yield followed a similar pattern as the fresh biomass yield. A decrease in yield with water stress treatments has also been observed in other studies, recommending a higher water availability for increased yield. A lower maximum allowable depletion (20% MAD) level resulted in better herbage yield of rose-scented geranium (*Pelargonium capitatum* x *P.radens*) [2]. In the first year of harvesting *P. sidoides* from the wild, the fresh biomass yield of 5.7 kg per 20 m^2^ plot with a mean population of 23.6 plants per plot was recorded [9], which translates to 11,800 plants ha^−1^ and yield of about 0.24 kg plant^−1^ or 2.8 t ha^−1^. The fresh biomass yield achieved by Lewu et al. [9] relates to about 16 t ha^−1^, which compares well with the yield recorded for the severe water stress treatment (70% ADL) in the current study. The current study suggests that irrigation at 30% ADL can increase the total biomass yield substantially (27.3 t ha^−1^), when compared to rainfed conditions in the wild. This is important since the leaves of *P. sidoides* showed some similar bioactivity as the roots, thus the whole biomass could possibly be used for medicinal purposes [8,24]. The reduction in yield under water stress could be due to stomatal closure, which decreased the rate of photosynthesis [4].

### 2.3. Root Yield

Fresh and dry root yields followed the same trend as the biomass yield, with water stress significantly reducing the root yield (Table 1), while N fertilization had no significant effect on root yield. The well-watered treatment resulted in significantly higher root yield values than the water-stressed treatments for both fresh and dry root yield, with no significant differences between the two water-stressed treatments. Water stress resulted in a more than 50% decrease in the mean fresh root yield of root chicory, compared to the control [25]. The dry root yield of *Thymus daenensis* was significantly higher in the non-stressed control (20% MAD), compared to the moderate and severely water-stressed treatments (50% and 80% MAD, respectively), while there were no significant differences between the two water-stressed treatments [26].

### 2.4. Metabolomic Analysis

**Figure 2:**
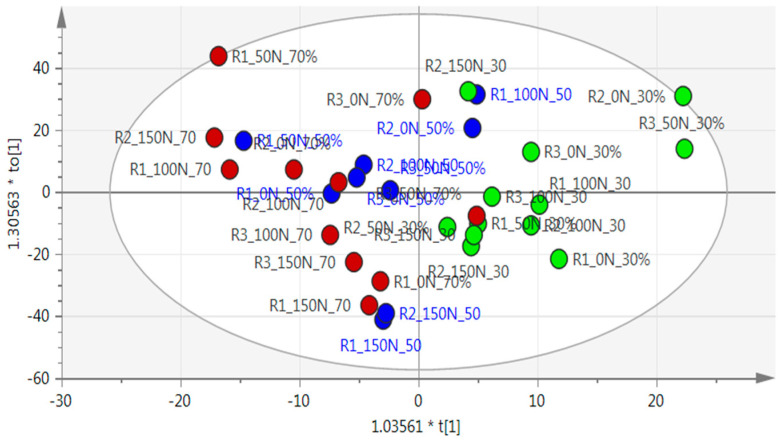
Orthogonal partial least square discriminatory analysis (OPLS-DA) results of *Pelargonium sidoides* on nuclear magnetic resonance (NMR) spectra, with the ellipse representing hoteling within 95% confidence. The well-watered treatment (30% allowable depletion level (ADL)) is represented by green dots, moderately stressed (50% ADL) by blue dots, and severely stressed (70% ADL) by red dots. R^2^X = 0.766 and Q^2^ = 0.101.

An unsupervised principal component analysis (PCA) showed no separation between treatments, and therefore, a supervised orthogonal partial least square discriminatory analysis (OPLS-DA) model was used to analyze the data, which separated the well-watered treatment (30% ADL) from the two water-stressed treatments (50% and 70% ADL), forming two groups (Figure 2). Data points of the severely stressed treatment (70% ADL) were generally clustered to the left (brown circle), with the moderately stressed treatment (50% ADL) (blue circle) more to the centre, indicating a gradual change from the well-watered treatment (30% ADL) on the right (green circle) to the severely stressed treatments on the left. The OPLS-DA statistical model showed goodness-of-fit (R^2^X = 0.766) with a lower predictability as the clusters were not clearly separated (Q^2^ = 0.101).

A contribution plot with subsequent use of databases such as the Human Metabolome database and Chenomx were used to annotate the major compounds in the plants subjected to the different treatments. The major compounds contributing to the separation were citric acid, glucose, sucrose, xylose, cis-aconitate, and trans-aconitate for the well-watered treatment (30% ADL), while asparagine, arginine, and 4-aminobutyrate were annotated for the water-stressed treatments (50% and 70% ADL); Table 2). Both the PCA and the OPLS-DA analysis on nitrogen treatments did not show separation between the treatments.

**Figure 3:**
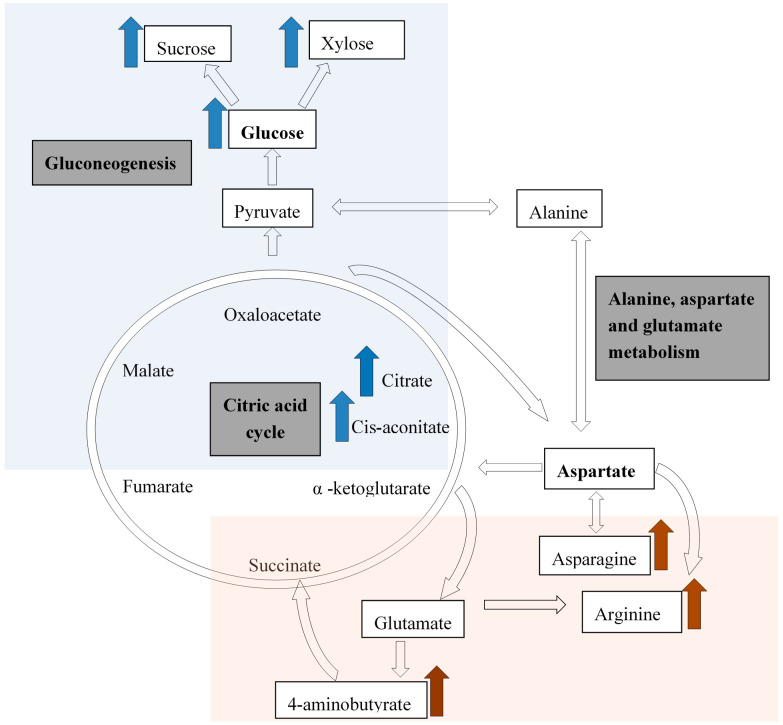
A simplified diagram indicating the citric acid cycle, the important metabolic pathways affected, and the resultant compounds that were increased during water-stressed (brown; 50% and 70% ADL) and well-watered conditions (blue; 30% ADL) adapted from previous researches [32,34], in *Pelargonium sidoides*.

The well-watered treatment (30% ADL) showed an increase in primary metabolites, especially the sugars linked to gluconeogenesis and the citrate cycle (Figure 3). Cis-aconitate is an intermediate in the citrate cycle and this explains the accumulation of citric acid in the well-watered treatment. As for the water-stressed treatments (50% and 70% ADL), the amino acids asparagine and arginine, as well as 4-aminobutyric acid increases, are all linked to the alanine, aspartate, and glutamate metabolism (Figure 3).

The accumulation of compounds such as free amino acids under drought conditions is responsible for osmotic adjustment in the plant [35]. An increase in concentrations of asparagine, with other free amino acids such as lysine, proline, leucine, histidine, glutamine, glycine, and threonine, in the leaves of barley (*Hordeum vulgare* L.) [36], potato (*Solanum tuberosum* L.) [37], and soybean (*Glycine max* L.) plants [38] exposed to water stress has been reported previously. Furthermore, sucrose and glucose content were reported to decrease in *Lupinus albus* L. plants exposed to water stress [39], an indication of the plants changing their metabolism from primary to secondary metabolites under water stress. Another free amino acid, a non-protein amino acid, 4-aminobutyrate, which is also known as GABA, was present in higher concentrations in the water-stressed treatments. GABA has been reported to be synthesized in response to water stress, amongst other stress factors [40], where it plays a role in stomatal closure through regulation of aluminum-activated malate transporter (ALMT) present in the guard cells of the stomata [41]. The accumulation of GABA is involved in enhancing stress resistance and in physiological responses, as an endogenous signaling molecule [42,43].

Endogenous GABA metabolism is mainly formed from glutamate by the activity of the cytosolic enzyme glutamate decarboxylase (GAD) [43], whereas asparagine is synthesized from glutamate and aspartate by the glutamine-dependent asparagine synthetase [44,45]. Free amino acids such as asparagine accumulate in plant tissues during water stress due to reductions in protein synthesis and an increase in hydrolysis [42]. Arginine is synthesized from ornithine, which in turn is synthesized from glutamate, by enzymes of the linear “arginine pathway” [44]. Arginine is linked to the biosynthesis of signaling molecules and is a precursor of proline, which maintains and improves the water status of plants during water stress [44,46].

### 2.5. High-Performance Liquid Chromatography (HPLC) Analysis of Selected Coumarins

Nitrogen and water stress had no significant effect on the *P. sidoides* root content of the coumarins: esculin, scopoletin, and umckalin (Table 3). However, the current study showed that cultivation could increase the umckalin content of *P. sidoides* roots, compared to the reported concentration of 67 ± 18 to 94 ± 13 μg g^−1^ (6.7 to 9.4 mg 100 g^−1^) from wild-harvested roots [5]. White et al. [5] further reported that under cultivation, water stress had no significant effect on root umckalin concentration; although in the wild, the highest umckalin concentration was found in roots collected from low rainfall areas. In the current study, only a slight (but non-significant) increase could be observed with lower water availability, although higher N application levels had the opposite effect on umckalin. For scopoletin, an increase in N levels slightly increased the compound levels, whereas water depletion resulted in a slight decrease. Different environmental factors, and the level of stress created, may, therefore, affect the production of these compounds in various ways with complex metabolic regulation. The resultant levels of production in the field would, therefore, be a representation of the various internal and external factors that regulate compound production [47]. This could mean that cultivation does not affect the medicinal value of *P. sidoides* roots with mild treatment conditions, but that more extreme conditions might result in dramatic changes of medicinal compounds, as is often found in the wild.

## 3. Materials and Methods 

### 3.1. Study Area 

A rainshelter field trial was conducted at the Agricultural Research Council, Roodeplaat-Vegetable and Ornamental Plants (ARC-VOP), Pretoria, South Africa (25°59′ S; 28°35′ E and 1200 masl). The rainshelter is designed to automatically open when there is no rain and close during a rainfall event, thus excluding interference of rainfall from the experiment.

The physical and chemical properties of the soil at the experimental site are as indicated by Mofokeng et al. [4]. The soil texture in the effective rooting depth (top 400 mm) was a sandy clay loam, with 16% to 22% clay content.

### 3.2. Plant Material and Trial Design 

*Pelargonium sidoides* stock plants were acquired from a nursery at the Golden Gate Highlands National Park, in the Free State Province of South Africa, and grown under a shade-net (grey, 40% shade effect) structure at ARC-VOP. Root cuttings taken from the mother plants, were rooted for four months and transplanted under the rainshelter. They were further established for four months before treatment commencement and harvested after six months. The trial was laid out as a randomized complete block design with three replicates and it was a 3 × 4 (irrigation levels × N levels) factorial experiment. Each treatment plot was 4.5 m^2^ in size, with a plant spacing of 0.5 × 0.3 m (66,666 plants ha^−1^).

### 3.3. Water and Nitrogen Treatments 

Irrigation treatments based on allowable depletion levels (ADL) of plant available water (PAW) from an effective rooting depth (ERD) of 400 mm, were 30% ADL (well-watered treatment), 50% ADL (moderately stressed treatment), and 70% ADL (severely stressed treatment). These predetermined percentages of PAW were depleted from the ERD before refilling the soil profile back to field capacity through irrigation. A neutron probe (Waterman, Probe Version 1.6, 2005, Geotech, SA) instrument was pre-calibrated for the trial site and calibration functions per 0.2 m of soil layer were developed following an established method [48]. Evapotranspiration (ET) was calculated for the growth period as:

ET = I ± ΔSWC (1)

where I and ΔSWC represent the irrigation water applied (mm) and change in soil water content (soil water content at the beginning of experiment minus soil water content at harvesting), respectively. Rainfall, runoff, and drainage were presumed to be zero. A computerized drip irrigation system (NETAFIM, South Africa) with a discharge rate of 2 L/h at a maximum pressure of 270 kPa was used for irrigation.

The following rates of N were applied as treatments: Control (0), 50, 100, and 150 kg ha^−1^ N. The N source used was limestone ammonium nitrate (LAN, 28% N), applied in two split applications of 50% each. The first N application was at four months after planting, while the second application was at eight weeks after the first application. Potassium (K) and phosphorus (P) were applied as a base application five days after planting, based on the soil nutrient status and estimated nutrient requirements of rose-scented geranium [23], since there were no fertilizer guidelines available for *P. sidoides*. Potassium (K) was applied as potassium chloride (50% K) at a rate of 110 kg ha^−1^ K, and P was applied in the form of single-super phosphate (11% P) at a rate of 30 kg ha^−1^ P once-off before treatment application.

### 3.4. Yield Data Collection and Statistical Analysis

Total fresh biomass and root yields were determined by weighing the whole plant (the aboveground parts and the roots) first, and then roots only, on a field scale (Model W113 platform digital scale, Richter Scale, Pretoria, Gauteng, South Africa) after harvesting.

The average dry matter content was calculated as follows:

(2) $$\mathrm{DMC}=\frac{\mathrm{TDM}}{\mathrm{TFM}}\times 100$$

where DMC is the dry matter content, TDM is the total dry mass, and TFM is the total fresh mass.

Data were subjected to two-way analysis of variance (ANOVA) using *GenStat*^®^ version 11.1. Treatment means were separated using Fisher’s protected *t*-test for least significant differences (LSD) at 5% level of significance.

### 3.5. Metabolomic Analysis 

#### 3.5.1. Sample Preparation and Extraction Method 

After harvesting the plants, roots were chopped into 2 mm pieces and oven-dried (Economy oven, 620 digital, Labotec, Midrand, Gauteng, South Africa) at 70 °C for 48 h. A powdered sample of 50 mg per treatment was weighed out in 2 mL Eppendorf tubes for extraction and analysis. Thereafter, 0.75 mL of CH_3_OH-d4 and potassium dihydrogen phosphate (KH_2_PO_4_), respectively, buffered in deuterium water (D_2_O) (pH 6.0) containing 0.1% (*w*/*w*) TSP (trimethylsilylpropionic acid sodium salt) were added to the samples. The mixture was vortexed at room temperature for 1 min, ultrasonicated for 20 min, and then centrifuged for another 20 min (10,000 rpm). The supernatant from each tube was transferred to a 5 mm nuclear magnetic resonance (NMR) tube (Norell, Sigma-Aldrich, Kempton Park, Gauteng, South Africa) for analysis.

#### 3.5.2. Data Acquisition/Sample Analysis 

NMR spectral data were obtained using a 600 MHz ^1^HNMR spectrometer (Varian Inc., Palo Alto, CA, USA), with 32 scans recorded and PRESAT setting to reduce the water peak. A description of how the NMR characterizes frequencies or chemical shifts of detected nuclei has been mentioned previously [49,50]. The phasing and baseline corrections were conducted using the MestReNova software (9.0.1, Mestrelab Research, Santiago de Compostela, Spain), with consistent settings for all sample spectra. The chemical shift ranges of δ 4.70–4.90 and δ 3.23–3.36, representing water and methanol, respectively [51], were excluded, while the remaining regions were normalized for further analysis.

#### 3.5.3. Data Mining and Processing 

The MestReNova software was further used for bucketing of NMR spectra. The NMR regions were divided into 0.04 ppm bins, resulting in 249 integrated regions. Thereafter, a multivariate data analysis (MVDA) was performed by the principal component analysis (PCA) and orthogonal partial least square discriminatory analysis (OPLS-DA). This was performed with the SIMCA-P software (13.0, Umetrics, Umeå, Sweden) using the Pareto scaling method. Scatter score plots from the PCA, which is an unsupervised analysis, were constructed to identify and evaluate groupings, trends, and strong outliers [51]. The second phase of analysis, the OPLS-DA, is a supervised pattern recognition method of which the main purpose is to separate the systematic variation in the X-matrix into two parts, with one part linearly related to the Y-matrix and one that is unrelated to the Y-matrix [52]. Contribution plots were constructed to identify important NMR regions, contributing to the separation of samples into the different clusters.

#### 3.5.4. Annotation 

The human metabolome database [53], Chenomx [54] databases, and other relevant references were used for annotation of compounds that were responsible for separations between treatment samples.

### 3.6. HPLC Analysis of Selected Coumarins

*Pelargonium sidoides* roots were analyzed for three main coumarins, which are esculin, scopoletin, and umckalin. The analysis was carried out at the ARC Analytical Laboratory, Pretoria, South Africa using adapted methods [5,55]. Following extraction of dry root biomass using 15 mL/g of methanol with sonication for 30 min, the filtered extract (20 µL) was injected into an HPLC equipped with a photodiode array detector (PDA) (Shimadzu, Kyoto, Japan). Chromatographic separation was carried out using a Luna^®^ 5 μm C_18_ 100 Å LC column (Phenomenex, Torrance, CA, USA). The column temperature was maintained at 35 °C. An isocratic mobile phase containing methanol:water:formic acid (30%:70%:0.1%) was used at a 1 mL/min flow rate. Peak identification and sample quantification were achieved using esculin, scopoletin, and umckalin (Sigma-Aldrich, Kempton Park, South Africa) concentrations used to plot the calibration curve.

## 4. Conclusions

In this study, the fresh biomass yield of *P. sidoides* was significantly increased with the application of N (50–100 kg ha^−1^) and sufficient water application (30% ADL). The study further showed that irrigation could increase the root yield of *P. sidoides* significantly, while N levels did not have a significant effect on root yield, although an increase in total biomass was observed. The metabolomics analysis showed that water stress induced the synthesis of metabolites such as arginine, aminobutyrate, and asparagine to mitigate the stress condition, whereas under no water stress primary metabolites such as sucrose, xylose, citric acid, as well as cis- and trans-aconitate, were synthesized for growth and increased yields. Therefore, it is proposed that there is a shift from the glycolysis/gluconeogenesis metabolism to the alanine, aspartate, and glutamine metabolism in water-stressed conditions to protect the plant under water deficit conditions. This could result in a higher uptake of nitrogen to supply the higher demand for N-based compounds in the alanine, aspartate, and glutamate metabolic pathway and not for primary growth. Furthermore, *P. siodides* can be successfully cultivated to reduce harvesting pressure on wild populations since the concentrations of esculin, scopoletin, and umckalin were not affected by irrigation and soil N amendment. The increased plant population under cultivation with well-watered conditions can increase yield significantly, compared to rainfed conditions in the wild, thus providing an opportunity for sustainable production and increased income generation for the rural poor communities providing plant material for the informal as well as commercial markets.

## Figures and Tables

**Table 1:** Total irrigation, evapotranspiration, yield, and root dry matter content of *Pelargonium sidoides* under well-watered and water-stressed conditions.

Water Depletion Level	Irrigation Water Applied	Evapotranspiration (ET)	Total Fresh Biomass Yield	Total Dry Biomass Yield	Fresh Root Yield	Dry Root Yield	Average Dry Matter Content
% ADL *	mm		t ha^−1^				%
30	466	431	27.30 ^a^	7.43 ^a^	19.77 ^a^	3.58 ^a^	27.24 ^a^
50	307	264	21.15 ^b^	5.20 ^b^	14.52 ^b^	2.51 ^b^	24.58 ^b^
70	256	237	19.22 ^b^	4.92 ^b^	14.05 ^b^	2.15 ^b^	25.57 ^ab^
LSD_0.05_	-	-	3.67	1.15	2.81	0.59	1.93

* ADL = Allowable depletion level; values in the same column followed by the same letters do not differ significantly (ANOVA test, *p* > 0.05).

**Table 2:** Specific NMR regions and annotated compounds, which contributed to the separation of the well-watered treatment (30% ADL) from the water-stressed treatments (50% and 70% ADL) as related to the contribution plots.

Treatment	1H-NMR Chemical Shifts (ppm)	Reference Chemical Shifts (ppm)	References	Chenomx	Compound
Well-watered (30% ADL)					
	2.52	2.51	[27,28]	2.55	Citric acid
	2.68	2.66	[27,28]	2.70	Citric acid
	3.17	3.11	[28]	3.18	Cis-aconitate
	3.45	3.95	[29]	4.36	Trans-aconitate
	4.55	4.62	[30]	4.57	Xylose
	5.15	5.22	[30]	5.19	Xylose
	5.44	5.40	[27]	5.4	Sucrose
	5.96	5.72	[28]	5.95	Cis-aconitate
	6.64	6.92	[29]	6.60	Trans-aconitate
Water-stressed (50% and 70% ADL)					
	1.64–1.72	1.66	[31]	1.61–1.75	Arginine
	1.88	1.91	[31]	1.89	Arginine
	1.88	1.90	[32]	1.88	4-aminobutyrate
	2.28	2.30	[33]	2.29	4-aminobutyrate
	2.82		[31]	2.85	Asparagine
	2.92		[31]	2.94	Asparagine
	2.99	3.02	[33]	3.00	4-aminobutyrate
	3.22		[31]	3.23	Arginine
	3.76		[31]	3.76	Arginine
	3.95	4.00	[31]	3.99	Asparagine

**Table 3:** Esculin, scopoletin, and umckalin content of *Pelargonium sidoides* roots as affected by nitrogen and water treatments.

Treatments	Esculin	Scopoletin	Umckalin
N * (kg ha^−1^)	**mg 100 g^−1^**		
0	27.6	3.4	42.5
50	29.5	3.5	45.3
100	24.9	4.5	38.3
150	25.4	4.8	39.0
ADL * (% PAW)	**mg 100 g^−1^**		
30	26.2	4.2	40.3
50	26.4	4.4	40.6
70	27.9	3.6	42.9
N x ADL	NS	NS	NS

* N = nitrogen, ADL = allowable depletion level, PAW = plant available water.
